# *Dual-Use and Trustworthy*? A Mixed Methods Analysis of AI Diffusion Between Civilian and Defense R&D

**DOI:** 10.1007/s11948-022-00364-7

**Published:** 2022-03-08

**Authors:** Stefka Schmid, Thea Riebe, Christian Reuter

**Affiliations:** Science and Technology for Peace and Security (PEASEC), Technische Universität Darmstadt, Pankratiusstraße 2, 64289 Darmstadt, Germany

**Keywords:** Artificial intelligence, Trustworthy AI, Values, Dual-use, Technological innovation policy, Network analysis

## Abstract

Artificial Intelligence (AI) seems to be impacting all industry sectors, while becoming a motor for innovation. The diffusion of AI from the civilian sector to the defense sector, and AI’s dual-use potential has drawn attention from security and ethics scholars. With the publication of the ethical guideline *Trustworthy AI* by the European Union (EU)*,* normative questions on the application of AI have been further evaluated. In order to draw conclusions on *Trustworthy AI* as a point of reference for responsible research and development (R&D), we approach the diffusion of AI across both civilian and military spheres in the EU. We capture the extent of technological diffusion and derive European and German patent citation networks. Both networks indicate a low degree of diffusion of AI between civilian and defense sectors. A qualitative investigation of project descriptions of a research institute’s work in both civilian and military fields shows that military AI applications stress accuracy or robustness, while civilian AI reflects a focus on human-centric values. Our work represents a first approach by linking processes of technology diffusion with normative evaluations of R&D.

**Supplementary Information:**

The online version contains supplementary material available at 10.1007/s11948-022-00364-7.

## Introduction

General consensus among ethics researchers underscores that as technologies based on Artificial Intelligence (AI) shape many aspects of our daily lives, necessary steps to be taken in technology development should include the assessment of risks and the implementation of safeguarding principles (Floridi et al., 2018; Taebi et al., 2019). AI is a general-purpose technology with manifold applications (Agrawal et al., 2018), and is considered a driver in emerging security-relevant technologies (Favaro, 2021). Further, China and the USA have joined the “global AI arms race” (Pecotic, 2019), indicating that they are ready to use AI for their military advantage. The prospect of proliferating autonomous weapon systems has not only convinced China and the USA but has also led other states to reevaluate their military advantage (Riebe et al., 2020). These innovations are often developed in the private sector, increasingly permeate social spheres, and have a high dual-use potential (Meunier & Bellais, 2019).

Accurately assessing risks of a dual-use emerging technology is challenging. The technology might develop in unprecedented ways, it might be used by hostile actors or accidentally cause harm. Therefore, understanding the diffusion of innovations is a decisive factor in the development of tailored risk assessment, governance measures, and opportunities of intervention regarding unintended and unexpected outcomes of emerging technologies (Tucker, 2012; Winfield & Jirotka, 2018). Regarding AI, civilian actors appear to be more engaged in Research and Development (R&D) for commercial end-use than actors in the defense sector. This suggests that directions and centralities of technology diffusion may have changed towards a stronger use of commercial innovation by defense firms (Acosta et al., 2019; Reppy, 2006; Shields, 2018). Approaching the diffusion of AI in European civilian and defense industries and its implications for responsible R&D, we pose the following question: *To what extent does AI diffusion occur in the EU and which patterns does it follow?*

We approach AI as a dual-use technology empirically and capture indications of envisaged trustworthiness in recent R&D as well. We investigate not only the extent of AI diffusion, which may already imply (ir)responsible R&D, but also norms that are diffused across civilian and military fields as well as normative patterns of AI R&D which may be indicated by values specific to the field of application (e.g., robustness for military applications vs. explainability for civilian applications).

Diffusion between military and civilian spheres implies that ethical guides such as the EU’s *Trustworthy AI* should consider the values of both military and civilian AI. The number of weaponry patents building on AI (G06N) patents is a measure of diffusion between spheres, as well as knowledge transfers between companies. Responsible R&D is characterized by awareness of technological development, and identification and regulation of unintended developments. Our mixed-methods approach draws on a combination of patent citation network analysis and qualitative content analysis. The quantitative analysis of AI diffusion is based on patents from EU member countries, which as such are commonly studied to approach innovations and knowledge transfers (Lupu et al., 2011). The qualitative content analysis, capturing specific values that are translated into military and civilian AI applications, focuses on projects of a German research institute dedicated to dual-use research (Fraunhofer IOSB, 2020). After presenting related work, as well as our methodological approach, we proceed to outline our findings. These are subsequently discussed regarding dual-use assessment and with reference to *Trustworthy AI*, which represents an approach of responsible R&D of AI, followed by a conclusion.

## Related Work and Theoretical Background

### Responsible R&D of Dual-Use Technologies

Commercial dual-use technologies have been discussed as a security matter and issue of risk assessment (Harris, 2016; Tucker, 2012). Research has examined the impact of defense innovations on civilian and commercial end-use, such as the invention of the internet (Mowery & Simcoe, 2002). By highlighting high-risk scenarios that do not impact military but rather civilian actors, the conception of dual-use technology has recently shifted towards being framed based on their socially “beneficial” or “harmful” (Brundage et al., 2018; Oltmann, 2015) or “good” and “malicious” purposes (Floridi et al., 2018). Recent understandings mainly focus on such purposes and (non-state vs. state) actors. Accordingly and focused on the character of the item only, Forge (2010) distinguishes between artefacts that are either purpose-built or improvised weapons. These considerations have prompted normatively oriented debates about dual-use and how to assess risks of emerging technologies while researchers and developers lack knowledge on future use and deployment of technologies (Grunwald, 2020). Our approach to capture AI R&D considers these various understandings and aims to set the foundation for a responsible assessment of dual-use research of concern (Evans, 2014; Riebe & Reuter, 2019).

The European patent network of AI inventions mainly considers whether such an invention belongs to the patent classification of weaponry (F41, F42). As this classification does not, however, take the context in which such inventions might be developed into consideration, we specifically take actors’ economic activity in the defense industry into account to determine either civilian or military use. We also follow this broader view on dual-use technology as applied both for defense and civilian reasons (by respective actors) when conducting the qualitative analysis of a research institute’s knowledge production. Thereby, we look for values of *Trustworthy AI* which may or may not be apparent in military and civilian applications and thus synthesize the assessment of dual-use technology with more recent, general ethical requirements. Determining the technology-specific characteristics of dual-use early in the process of R&D is part of the iterative process of technology assessment (TA) to further establish measures and to balance “risks and benefits” (Tucker, 2012).

### EU *Trustworthy AI* Principles

Trust and trustworthiness have previously been discussed focusing on interactions among both autonomous and human agents (Taddeo, 2010; Wagner & Arkin, 2011). Trustworthiness is understood as “the guarantee required by the trustor that the trustee will act as it is expected to do without any supervision” (Taddeo, 2010). Further, trust is defined as in the following:

If the trustor chooses to achieve its goal by the action performed by the trustee, and if the trustor considers the trustee a trustworthy agent, then the relation has the property of being advantageous for the trustor. Such a property […] is called trust (Taddeo, 2010).

Concerning AI’s potential to secure systems from cyberattacks, Taddeo et al. (2019) argue that trust is unwarranted due to vulnerabilities, while reliance on AI indicates “some form of control over the execution of a given task”. Tavani (2018) stresses that relational approaches, which are more interested in technology’s appearance to humans than its properties, may consider the diverse and diffuse relationships defining trust. People may forget that they are dealing with artificial agents (Taddeo, 2017), which is only remembered “when something goes (badly) wrong”. In this regard, the question has been raised whether artificial agents, including military drones, should imitate human characteristics like empathy or the feeling of guilt (Arkin et al., 2012). The research towards mimicking humans and human behavior has been criticized by Grodzinsky et al. (2011) as accurate identification of agents may determine trust. They further stress that self-modification of artificial agents poses high risks for public safety. Therefore, the loss of human control in interaction with artificial agents which mimic well-known human behavior may carry more risks than advantages for the trustor.

Public trust can be achieved through the establishment of ethical codes, responsible practices, and procedures that ensure ethically aligned governance of technology (Winfield & Jirotka, 2018). Nissenbaum (2001) has argued that trustworthiness is crucial for the acceptance of technology by referring to Luhmann’s (1979) understanding of trust as a “mechanism for reducing complexity”. As such Nissenbaum (2001) argues that trust allows for “creative, political, unusual, […] possibly profane, […] risky modes and activities” to flourish in a loosely secured cyberspace. Thereby, she emphasizes trust’s productive nature which allows for the adoption of AI in various fields of application (ibid.). While trust may facilitate procedures, substantial guides such as *Trustworthy AI* which formulate requirements do not simplify human engagement. Instead, they may indicate regulation (and securitization) efforts which allow for the establishment of trust in the first place.

While *Trustworthy AI* is one of the most important documents by the EU in this regard, overviews of institutional guidelines echo common vocabulary and the direction of recent guidelines. Roberts et al. (2021) investigate the 2017 “New generation artificial intelligence development plan” and highlight socio-political conditions which may have shaped China’s AI strategy. Although an important actor, China had at first only seldomly engaged in ethical debates regarding AI but is now propagating shared values of AI R&D, such as human well-being, fairness, and transparency (Roberts et al., 2021). Similar to other relevant actors in AI innovation, it has started to raise ethical questions about AI R&D. Thiebes et al. (2020) have compared current approaches of trustworthy AI, highlighting requirements like robustness, lawfulness, as well as various principles (e.g., beneficence), which summarize the core values of different ethical frameworks on AI (Hagendorff, 2020; Thiebes et al., 2020). Our work adopts this perspective on diffusion of AI by contextualizing it as a dual-use technology which is supposed to meet normative, albeit differently defined, criteria of trustworthiness.

The “Ethics Guidelines for *Trustworthy AI*” (European Commission, 2019) were published in 2019 and comprise legal, ethical, and technical pillars. While the expert group highlights the importance of the three pillars, the guideline itself heavily focuses on the second and third dimensions (European Commission, 2019). The identified values (see Table 1. “Appendix”) may be reflected at the institutional and technological level (European Commission, 2019). Representing norms, the document also provides a set of criteria for TA by developers and end-users (European Commission, 2019). In our study, we include the most relevant values and summarize some of them thematically. For the analysis, we follow the Value-Sensitive Design (VSD) approach, which is interested in deriving values from human-technology interaction^1^ (Cummings, 2006). The EU guideline deviates from a traditional understanding of dual-use and stresses the differentiation between beneficial and malicious use (European Commission, 2019), referring to a publication by Brundage et al. (2018). This corresponds to the recent R&D policy of the EU, aiming for synergies between civilian and military knowledge production and application (Edler & James, 2015; European Commission, 2015; Uttley, 2019).

### Knowledge Diffusion of AI

To capture AI development, political actors have conducted analyses relying on different measurements. This includes a focus on citations and keywords of patents and scientific literature as well as analysis of open source software. Insights into processes as well as spatial and temporal frames of R&D have become crucial for governments which are engaged in funding AI innovation (Baruffaldi et al., 2020). Patent data serves as an indicator for applied knowledge or technological innovation (Lupu et al., 2011; Meunier & Bellais, 2019), as patents demonstrate intellectual property of inventions while citation networks indicate diffusion of purposeful, codified knowledge (Liu et al., 2019; Pereira & Quoniam, 2017). It should also be noted that AI is a contentious term contouring different techniques (Cady, 2017; Goodfellow et al., 2016; Klinger et al., 2018).

Interested in the diffusion of AI in both the EU’s civilian and defense spheres, our work is inspired by the extensive body of patent analyses and thereby adopts a relatively classic approach of innovation research as a first step. Zambetti et al. (2018) conducted a patent network analysis focusing on machine learning (ML) and AI-related techniques to examine relevant industrial players. They show how ML-related technologies are mostly driven by software companies but also spread to other sectors. This has led to the 4th industrial revolution, as companies can invest in capitalizing their data and analytic capabilities (ibid.). However, these contributions do not distinguish between defense and civilian industries, and either omit or do not entirely consider the ethical questions of AI diffusion.

Other patent analyses interested in defense economics or arms control have specifically concentrated on warfare technologies, such as drones, ammunition, or radar technology, and looked at the extent of diffusion or tested explanatory hypotheses on the impact of defense R&D funding (Acosta et al., 2011, 2013, 2017, 2019; Meunier & Bellais, 2019; Schmid, 2017). Our study on AI diffusion ties in with existing works on dual-use technology and comprises patent analysis. However, as other arenas of knowledge transfer need to be considered as well, we accompany this approach with a qualitative analysis of knowledge diffusion, referring to the EU guide *Trustworthy AI*.

## Research Design

### Patent Analysis: The Case of AI

AI may be part of computer-implemented inventions (Okakita, 2019) and thereby fall under a patentable subject matter, which can be distinguished from discoveries, scientific theories, mathematical methods and “mental processes” by its “technical character”. This implies a “‘further technical effect’, which goes beyond the ‘normal’ ‘physical’ interactions between the program (software) and the computer (hardware)” (European Patent Office, 2021b; Okakita, 2019). This understanding is prevalent across patent offices, such as within the European Patent Organization (EPO), and the US Patent and Trademark Office (USTPO) (Okakita, 2019). Further, inventions must be novel and applicable to a specific industrial area (WIPO, 2019). This includes, e.g., “the use of a neural network in a heart-monitoring apparatus for the purpose of identifying irregular heartbeats” or new classification systems (Okakita, 2019). The standards for patent eligibility might also change due to the rise of AI and the need for patent regulation to adopt to them. In 2018, the EPO published a new guideline on ML and AI, which was criticized as it did not acknowledge AI and ML the same way as other highly abstract areas, such as encryption (European Patent Office, 2021a; Korenberg & Hamer, 2018). In the context of military applications, due to their confidential nature, innovations may not always be published as patents, while economic disadvantages may prevail as well (Schmid, 2017; Urquhart & Sullivan, 2020).

In our research design, we follow existing studies which have focused AI’s patentability and its inventiveness (Okakita, 2019). We therefore focus on the CPC class G06N^2^ of “systems based on special computational models”, with sub-classes like “artificial life” or “computer systems based on biological models” (CPC, 2019) and build on previous work which has focused more broadly on G06 patents in their investigation of innovation spillovers regarding unmanned aerial vehicles (UAVs) (Kim et al., 2016). Here, patent information, comprising publication date, country, back and forward citations, applicants, and thematic classifications, provide the foundation for quantitative investigation of cross-country knowledge diffusion. The patent analysis also includes an exploration of company networks. It focuses on German patents, with Germany being an important market for both AI and weaponry, reflected by a large German share of the European sample. We thus focus on the most populated and economically strongest country in the EU. While the EU constitutes an important norm-setting actor, Germany plays an important role in the EU as a civilian power (Cath, 2018; Koenig, 2020).

### Research Bodies: Arenas of Knowledge Diffusion

Since the focus of our quantitative analysis lies on company networks in Germany and German patents, and considering that diffusion may also take place without patenting inventions, our qualitative analysis focuses on the normative patterns of AI diffusion on research projects of the German research institute Fraunhofer Institute of Optronics, System Technologies and Image Exploitation IOSB. The institute belongs to a prestigious group of Fraunhofer institutes and is one of the main scientific actors regarding research on military applications in Germany (Fraunhofer IOSB, 2020; German Federal Ministry of Defense, 2017). Fraunhofer IOSB encompasses both civilian and military business units (Fraunhofer IOSB, 2020), in which relevant knowledge for AI applications is produced. The text documents selected for this analysis imply inter-organizational knowledge transfers between the Fraunhofer IOSB, the German Ministry of Defense, and Armed Forces. They reflect knowledge of specific military AI applications, produced in close cooperation with military actors and sometimes transferred intra-organizationally (Fraunhofer IOSB, 2018). This allows a comparison of projects regarding both civilian and military applications of AI.

### Data Collection

**Fig. 1:**
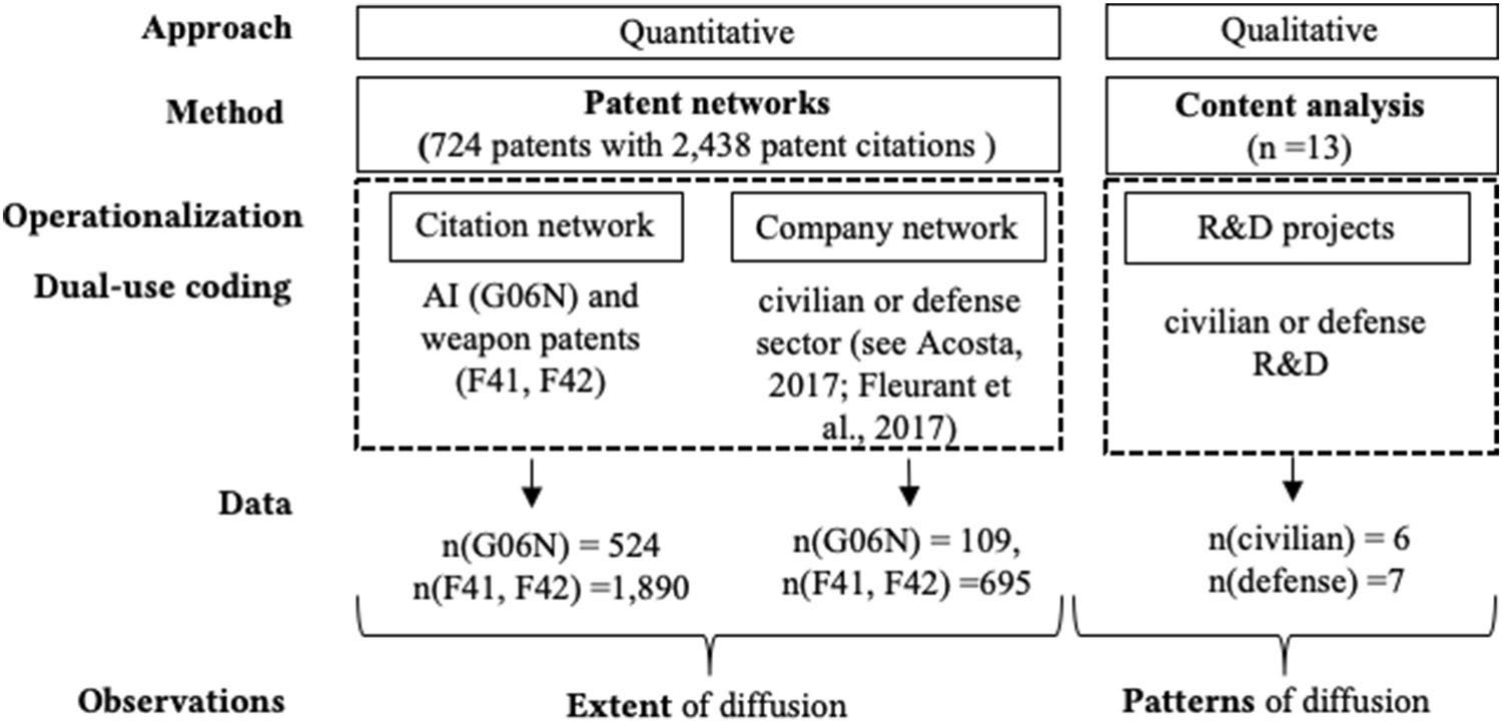
Mixed-methods research design, data and observations

To conduct the statistical part of the analysis, we retrieved data from the EU patent database Espacenet. Interested in the recent developments of diffusion, we limited our search to patents from January 1, 2008 to June 1, 2018. We collected data based on all country codes of EU member states and the patent classifications of AI (G06N) as well as ammunition and weaponry (F41, F42). This resulted in a data set compromising 5,365 patents, with weaponry-related patents representing military and AI patents constituting civilian inventions. The sample was then reduced to patents that cited other patents, reducing the sample to 724 patents with a total of 2438 patent citations (see Fig. 1). The second step of the analysis focused on the specific type of AI diffusion and allowed insights into the R&D of AI applications. We selected 13 documents, all of which were freely accessible online via the Fraunhofer IOSB homepage. The corpus of different types of documents reflects both military and civilian applications, as well as different conceptual and technological levels of detail, ranging from web pages with project descriptions, flyers to scientific publications of all business areas (see Table 2, “Appendix”). They allow a deeper and balanced, yet not representative insight into R&D of AI applications.

### Data Analysis

For the data analysis, we chose a mixed-methods approach (see Fig. 1). This two-step analysis can shed light on the various fractions of how diffusion of AI has taken place, including the patterns it follows. We conducted descriptive statistics in Microsoft Excel. Further, we constructed two networks in RStudio, both based on our data set, one focusing on links between patent classifications and the other among German patent applicants. Our work follows the logic of patent network analysis, where relevant entities form nodes connected by patent citations. For the qualitative analysis, we performed a content analysis (Flick, 2014; Gray et al., 2007) and relied on RQDA (Ronggui, 2019). The code categories and (sub-)codes were developed abductively, inspired by the EU’s formulation of *Trustworthy AI* and related scholarly works as well as based on the empirical material of Fraunhofer IOSB. Frequencies of words were examined through text mining (see supplementary material Figure A, Table A). While this qualitative part of our work does not constitute a representative study and only specifically refers to a few documents, we used all selected documents for the quantification of results.

## Analysis

### Quantitative Analysis: Patent Citation Networks

Our study of patent information comprises the analysis of a patent citation network based on the patent groups of weaponry and AI and a subsequent focus on German patents and relationships between involved companies.

### European Network Based on Patent Classes

We assumed CPC classes^3^ to constitute nodes, while edges were determined by patent citations. Our data set contains 2438 patent citations, including 24 of unknown origin. While 524 patents are cited by AI patents, 1890 patents are cited by weaponry patents. Most of the patents referred to patents of the same CPC class (see supplementary material Figure B). Since we are particularly interested in linkages representing knowledge transfers across the fields of weapons and ammunition (F41, F42) and special computational systems (G06N), we note that there is no such transfer-representative patent citation. Among AI patents, however, 14.6% of the patents cited other G06N patents, constituting the biggest group comparatively. Looking at weaponry patents, citational links exist frequently to other weapons and ammunition technologies. For example, F42B patents citing other F42B patents make up the largest share of weaponry patents and their citations (23.6%).

Considering responsible R&D as illustrated by the EU’s guideline *Trustworthy AI*, our results do not point into the direction of frequent and widespread knowledge transfer among civilian and defense actors through their technologies. Consequently, we could not find evidence supporting the hypothesis of emerging technologies such as AI being applied primarily for civilian purposes and subsequently for military purposes (Verbruggen, 2019). Focusing on German patents allows diving into company linkages representing knowledge transfers within the national network.

### The German Company Network

Taking a closer look at German patents, knowledge transfers between defense and civilian industries can be approached apart from solely relying on the CPC system. We assume companies as applicants of patents to be the driving actors for inventions. Therefore, we reconfigured a network based on companies as nodes, while one company is linked to another company by citing at least one patent of the respective applicant. In the interests of precision, we concentrated on German patents and their cited patents from companies occurring more than once in the sample.

An operationalization approach guided less by classifications assumes that certain companies might remain very active in the defense industry, despite not formally considering filing a patent application. Following the Stockholm International Peace Research Institute’s (SIPRI) Top 100 list of companies in arms sales, these companies can be categorized as part of the defense industrial sector (Acosta et al., 2017; Fleurant et al., 2017). Even though many of the companies are mainly active in other industrial sectors such as aviation and aerospace, producing revenue of more than $840 m. per year in the defense sector allows these companies to specialize in R&D of defense technology and consequently compete with other actors in this regard (Riebe et al., 2021). Other companies are labeled as a military company if their share of arms sales represents more than 50% of total sales. In case no figures are available and qualitative analysis is applied to determine whether most of the produced^4^ goods’ business areas are part of the defense industry. This allows to re-illustrate German companies’ relationships in form of a network (see supplementary material Figure C).

**Fig. 2:**
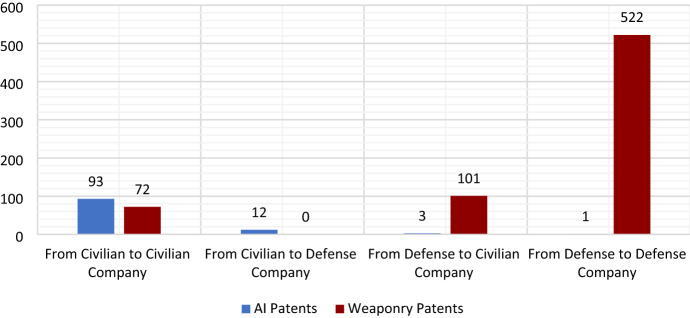
Knowledge transfers among companies by patent activities according to their main business areas, n = 804

**Fig. 3:**
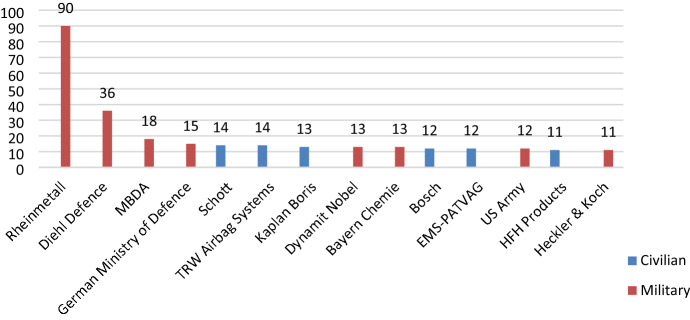
Most important actors of the company network, with at least ten connections to other entities

The analysis of the patent citation shows almost no diffusion between civilian and military companies. Most AI patents are based on interactions between civilian entities, with only one citation pair among defense companies (Airbus citing Lockheed Martin). Additionally, there are three patent pairs with civilian actors that cite patents from defense companies. Another 72 pairs of citing and cited weaponry patents are from companies that usually produce for civilian markets (see Fig. 2, “Appendix”). The prevalence of defense actors is evident in the large number of weaponry patents. The most active applicants rely on actors of the same type or mostly on themselves. For example, Rheinmetall has cited 231 of its own patents (see Fig. 3, “Appendix”). The concept of *Trustworthy AI* generally highlights the importance of corporate ethical requirements and potentially unintended consequences (European Commission, 2019). However, it disregards the relevance of industrial civilian-defense sector ties or the dual-use activities of companies. Further, we shed light on research projects that entail the diffusion of AI across civilian and defense spheres. In contrast to the quantitative approach, the qualitative approach allows us to gain a more concrete picture of how AI diffusion takes place, illustrating which values of trustworthiness are incorporated into the technology and are revealed in human interactions with AI.

### Qualitative Analysis

#### Trustworthiness of Military AI Applications

Robustness, accuracy, and information quality seem to be apparent values which support *Trustworthy AI,* when considering military purposes. This does not mean that these norms are entirely absent when it comes to civilian AI applications. Instead, our analysis indicates that they are relatively more prevalent in the military context (see supplementary material Table A). Thus, as a value, *robustness* is comparatively more significant in the context of military (D11) applications, including resilience as an important standard (D10). Further, *accuracy* is particularly important in the context of military applications, including transparency on problems of inaccuracy:

Although the RMS [root mean square; author’s note] errors for building reconstruction […] indicate that our method provides reasonable geometrical *accuracies* (height error is the same as for single points if the parallax accuracy is about one image pixel), the results in building detection are *less precise.* (D12.; own emp.)

Similarly, the EU guideline stresses the importance of the technical values of robustness and accuracy. These relate to both safety and security, which are crucial in warfare scenarios. Military AI applications may support standards of *Trustworthy AI*, paying special attention to robustness and accuracy (European Commission, 2019) in more critical contexts. This reflects the potential to ensure security as proposed by the EU guideline (European Commission, 2019), while also indicating the technology’s possible normative ambiguity regarding general human and environmental well-being. *Information quality* has also been relatively more important for military (D1) applications. Given the high stake of a military operation, errors due to low information quality may have a greater impact on people, e.g., by mistaking civilian infrastructure for military bases or by falsely engaging civilians as combatants.

#### Trustworthiness of Civilian AI Applications

At the same time, there is a comparatively stronger interest in civilian projects in *awareness*, indicating the importance of capturing the environment in all its complexity. For example, SPARC, a project on autonomous driving in urban traffic, relies heavily on orientation in the context of moving and directing surrounding objects, opting for a “holistic representation” (D3), while at the same time training data is focused on “eventful […] and […] unique situations” (D13). Whether in terms of space, time, or speed, there is a strong reference to environmental information. This is surprising, as situational awareness is not only stressed by the EU (European Commission, 2019) but is mostly apparent in military contexts.

Overall, civilian applications emphasize the relevance of *explainability,* which is referred to as “retaining many of the advantages of variational trajectory optimization methods, in particular *expressiveness*” (D11; own emphasis). Others underline that “[t]he ability for humans to understand the reasoning process is essential to the presented case study” (D13). This highlights the ambivalence of explainability as a normative concept. While it may be defined as the ability to explain, interpretability, namely the ability to provide (grounds for) an interpretation, is often associated with the concept of explainability, as it is also the case in the *Trustworthy AI* guide (European Commission, 2019). This requirement for civilian applications may be plausible, should special attention be paid to a broader and more diverse group of end-users. This becomes particularly apparent considering that the project on autonomous driving in cities (D11) stresses explainability (or expressiveness) the most.

It should be noted that both *security and safety* were also qualitatively deduced regarding military applications, indicating human-centric approaches albeit in different terms. Human dignity, implying human-centric approaches, represents one of the core values of *Trustworthy AI* (European Commission, 2019). Such statements are more common in the context of civilian applications; as they apply AI applications that put focus on human reasoning, hand gestures, or the human body (see supplementary material Table A). Military applications accordingly reflect less interest in a precise analysis of the social or intimate environment. Yet, a strong focus on people’s movements or behavior does not necessarily imply the implementation of a human-centric AI in terms of human dignity or personal rights.

#### Diffused Values Across Civilian and Military Applications

Regardless of the field of application, the authors of scientific publications were *transparent* about procedural problems. In contrast, AI was depicted relatively flawless in online presentations of projects or product flyers. This may be due to the nature of scholarly debates, supporting values such as transparency (of problems). Problematic issues were not made transparent in shorter, more easily accessible online contributions, while such documents contained more direct references to economic merits. The European expert group’s guide would suggest presenting complex, inconvenient facts to a broader audience and allow for understandability independent from personal background (European Commission, 2019). Furthermore, the figurative alignment of AI and animal behavior became visible. AI projects were oriented towards phenomena in nature, for example in the development of “swarms” of UAVs or processing as in an “ant colony” (D10). AI was also designed to imitate the human essence. This is reflected in notions about the AI’s self and its abilities (see supplementary material Table A). *Trustworthy AI* refers to approaches such as values-by-design, implying a certain degree of technological agency (European Commission, 2019). However, Fraunhofer projects do not reflect the awareness of such interactional approaches or non-human agency. While projects indicate anthropomorphization of AI as well as bionic models, they do not guarantee trustworthiness based on environmental awareness.

## Discussion

### Implications for Dual-Use Assessment

The patent citation network analyses did not indicate direct diffusion of AI into patents for weapons and ammunition (F41, F42). This contradicts hypotheses stating that AI diffuses relatively easily from civilian to military industries due to its innovative and intangible nature (Acosta et al., 2019; Gill, 2019; Reppy, 2006; Shields, 2018). While inventions of weaponry mainly rely on other patents of the same field, they have also benefited from patents of civilian categories in the past. However, most of citations and cross references are found within the same patent category.

As pointed out in an interview with the Patent and Brands Center Rhein-Main, there is always the option of classified patenting (2019, personal communication). Looking at the patent networks, AI diffusion across defense and civilian fields is low and could only be observed within individual organizations. Drawing from this, TA, which aims at providing prospective knowledge for responsible R&D, should focus on other spaces of knowledge transfer among businesses and research bodies instead of patent regimes. In general, regulation through the publication of patents may generate of trust. While we do not share the dichotomous perspective of unregulated trust relationships vs. highly regulated ones (Nissenbaum, 2001), we follow the idea of trust allowing for “risky” modes of behavior. To create relationships based on trust, regulatory efforts such as TA that focus on the diffusion of foundational knowledge of research may be necessary in the first place. In this context, the case of Europe is very interesting as the EU tries to incentivize synergies between defense and civilian industries to increase competitiveness of the defense and security sector (Edler & James, 2015; European Commission, 2013; Uttley, 2019). At the same time, the EU has fostered research to monitor the diffusion of dual-use innovations, to understand the networks and technological developments.^5^ Dual-use research of concern has provided approaches of risk assessment for individual researchers and organizations (Evans, 2014; Tucker, 2012), such as raising awareness, defining norms and supporting public discourse on technology related risks and possible future developments of socio-technical systems (Grunwald, 2020). Coeckelbergh (2020) has developed the discussion of distributed responsibility further by discussing a relational framework, making AI experts responsible for risk communication. Winfield and Jirotka (2018) showed a framework for ethical governance of AI and robotics companies, in which a network of regulatory bodies, regulations, and verification work together to build public trust. However, the discourse on effective yet flexible regulations and norms is still ongoing. In the following, we consider implications for *Trustworthy AI* regarding dual-use research.

### Implications for *Trustworthy AI*

The parallel increase in scientific publications on AI (WIPO, 2019) allows to highlight an additional focus on innovation diffusion by knowledge transfers in applied research. In this regard, the patterns of values reflecting responsible R&D, i.e., *Trustworthy AI* may be identified depending on the specific field of application or diffused across technologies. While differentiating between beneficial and malicious usage of AI may prove valuable in assessing the societal impact of an application (Brundage et al., 2018), a stronger focus on AI as a dual-use technology applicable for both civilian and defense purposes allows considering applications that have a decisive impact on human life. Such applications include the automated surveillance and analysis of people to gain intelligence information as well as automated functions in armed systems to engage selected targets.

Design approaches referred to in the EU guide and other studies (European Commission, 2019; Umbrello & De Bellis, 2018) offer possibilities for appropriate implementation. As an umbrella organization of research institutes, Fraunhofer has incorporated interdisciplinary work (Marzi et al., 2018). However, concerning the studied research groups, the documents did not suggest room for a diverse discourse in favor of a *Trustworthy AI*, which would promote further deliberations (European Commission, 2019) on trust, the anthropomorphization of AI (Ryan, 2020), and general acceptance of AI technologies (Winfield & Jirotka, 2018). A transparency report, as suggested by Winfield and Jirotka (2018), could include a statement on results that may be difficult to interpret, as well as a reflection on institutional contexts and diverse societal effects of implementation. Additionally, the different approaches towards trust and moral decision-making by artificial agents (Arkin et al., 2012) may become an increasingly important issue for TA. *Trustworthy AI* might benefit from encouraging discussions about concrete procedures for fruitful interdisciplinary work and clarification of contextual conditions, such as economic competitiveness in the application of ethics. Interpreting the different values of civilian and military AI applications suggests that *Trustworthy AI* is more consistent when the diversity of contexts is included. As *Trustworthy AI* is considered a “horizontal foundation” to facilitate the development of trustworthy innovation, the EU suggested to add “sectorial” perspectives to adjust to the context-specificity of AI systems (European Commission, 2019). To assess the diffusion of innovation the context needs to be considered, while expanding the focus on related sectors. Prioritization of values differs regarding the context of application. This influences the diffusion of innovation as adjustments to other requirements have to be made but more significantly, as values are inscribed in the technology. While we illustrate how the prevalence of values may differ across fields of application, we do not propose that they are exclusive to specific sectors. Instead, our study proposes a vantage point for future research on norm emergence such as dual-use focused TA, potentially including stakeholder analysis.

Finally, our study indicates that some of the values are closely associated, such as explainability and interpretability or well-being, safety, and security. Thiebes et al. (2020) propose five principles of trustworthy AI, offering a synthesis of relevant values or requirements of ethical frameworks, such as in the EU guide (European Commission, 2019). While there is indeed common ground regarding relevant values that influence relationships of trust, our analysis emphasizes the importance of finding a common language and clarifying the existence of divergent understandings that may prevail across different national frameworks, albeit references to the same labels (Roberts et al., 2021).

### Limitations

As we focus specifically on AI patents, we did not include patents for advanced robotics of the class B64G 2001 (USPTO, 2019) or other commercial areas (e.g., aviation and aerospace), and therefore limited the sample to G06N patents. In addition, our sample only includes patents that cited at least one other patent, which is further limited by a focus on German patents for reasons of clarity. Even though the EU is one of the most active regions with regard to filing patents, especially in the AI field, many more patents are filed in Japan, the US, and China (Baruffaldi et al., 2020), thereby limiting the scope of this study and its implications to the EU with a focus on German R&D. Furthermore, certain innovations may be protected by secret patents and others may be subject to trade secrets or copyrights, or refrain from patent registration due to complicated analysis of territorial eligibility (Tiedrich et al., 2020). Companies may remain competitive, using Machine Learning as a service instead of developing their own applications (Guthrie, 2019).

## Conclusion

AI is seen as a general-purpose technology, and the study of the patterns of diffusion of innovation between civilian and defense applications is relevant not only for TA but also regarding normative concepts that influence the R&D of AI, such as *Trustworthy AI*. As a mixed method approach, we conducted a patent citation network analysis in the first step. Considering member states of the EU as well as defense and civilian contexts of application, this work studied innovation transfers between AI and weaponry patents and took company relations into account. While the patent citation network did not show any diffusion between weaponry patents and AI, the close-up on the German company network revealed that a few defense companies publish both AI and weaponry patents, which might also be due to their dual-use products. As the second part, the qualitative analysis of technology descriptions of both civilian and defense R&D projects of the Fraunhofer IOSB, allows reevaluating established measurements and playgrounds of technological diffusion. The diffusion of trustworthy AI norms between defense and civilian R&D projects revealed the hierarchical context-specific application of certain *Trustworthy AI* norms, such as robustness and accuracy for defense projects and explainability for civilian projects. While attention is paid to R&D of AI, both economically and politically, it is relevant to gain insight into this development and to establish methods for its tailored dual-use and risk assessment and awareness measures to prevent unintended outcomes (Tucker, 2012; Winfield & Jirotka, 2018). Advanced and further work may address the political context of *Trustworthy AI* and accompany EU strategies of fostering the development of dual-use technologies, with a focus on economic synergies (Edler & James, 2015).

### Electronic supplementary material

Below is the link to the electronic supplementary material.

Supplementary file 1 (PDF 171 kb)

Supplementary file 2 (PDF 254 kb)

Supplementary file 3 (PDF 174 kb)

Supplementary file 4 (PDF 156 kb)

## Appendix

See the Tables 1, 2 and Figs. 2, 3.

**Table 1:** Values that constitute Trustworthy AI according to the respective EU document (European Commission, 2019), non-exhaustive overview

Normative dimensions	Values
Lawfulness	EU primary law
	Secondary law
	UN human rights treaties, conventions of Council of Europe, EU member state laws
	Domain-specific rules
	Ensuring due process and equality before law
	Citizens’ rights
Robustness (socio-technical dimension)	Safety
	Security
	Offering alternatives
	Accuracy
	Reliability
	Reproducibility
Ethical dimension	Human autonomy, oversight
	Human centric view (choice), dignity
	Human rights and freedoms
	Explicability (incl. interpretability)
	Paying particular attention to vulnerable groups (historically disadvantaged groups, people with disabilities, children, unequal access to resources)
	Privacy, data governance
	Environmental and social well-being
	Competitiveness
	Accountability and responsibility
	Involvement of stakeholders in all steps
	Mindfulness of tensions (e.g., trade-off with accuracy)
	Working towards continuous improvement
	Trust
	Holistic approach
	Quality of service indicators (incl. traditional software metrics of functionality, i.e., usability, performance, maintainability)

**Table 2:** Overview of text documents used for the analysis (accessible via the Fraunhofer IOSB webpage and the related online library catalogue; at least one author of each publication is associated with the Fraunhofer IOSB)

Number	Document	Type	Field of application, topic
D1	“Business Area Artificial Intelligence and Autonomous Systems”	Webpage	Civilian
D2	“KonsensOP: Context-sensitive Assistance in Aware Op”	Webpage	Civilian, medicine
D3	“SPARC—Situation Prediction and Reaction Control: The SPARC-Concept for fully-automatic Driving”	Webpage	Civilian, driving
D4	“Business Area Defense: Facilities”	Webpage	Military
D5	“Business Area Defense: Overview”	Webpage	Military
D6	“Business Area Defense: Fields of Activity”	Webpage	Military
D7	“CSD (Coalition Shared Data) Server based on STANAG1 4559”	Product flyer	Military, information processing
D8	“PoET 2.0: Image-based Determination of Optimal Camouflage in Practice”	Article	Military
D9	“Situation Detection for an Interactive Assistance in Surgical Interventions Based on Dynamic Bayesian Networks”	Publication (by Philipp et al. 2016)	Civilian, medicine
D10	“Using Heterogeneous Multilevel Swarms of UAVs and High-Level Data Fusion to Support Situation Management in Surveillance Scenarios”	Publication (Bouvry et al. 2016)	Military, surveillance
D11	“A Tractable Interaction Model for Trajectory Planning in Automated Driving”	Publication (Ziehn et al. 2016)	Civilian, driving
D12	“Context-based Automatic Reconstruction and Texturing of 3D Urban Terrain for Quick-response Tasks”	Publication (Bulatov et al. 2014)	Military
D13	“Automatic Understanding of Group Behavior Using Fuzzy Temporal Logic”	Publication (Ijsselmuiden et al. 2014)	Civilian, surveillance

## Footnotes

1 For exemplifying works building on VSD in their analysis of military AI, see Umbrello (Umbrello, 2019; Umbrello & De Bellis, 2018) or Verdiesen (2017).

2 For example, an invention by Amazon Technologies, Inc. falls under the category G06N. Callari et al. (2021) present techniques for managing a group of autonomous vehicles (AVs) to perform delivery tasks and thereby also rely on other G06N patents.

3 Groups of patents were defined according to the Cooperative Patent Classification (CPC) system (European Commission 2019).

4 Following Riebe et al (2021), a company’s website and respective insight into its business areas, range of products, or alliance of cooperation partners proves helpful. If a company is not included in the SIRPI Top 100 arms selling companies list and most of its sales lies within the civilian industries, it is defined as “civilian”. An ideal type of a dual-use company is defined by equal (50:50) share of civilian and defense sales. While suppliers for military infrastructure like telecommunication may also have industrial contracts with national governments, we focused on companies that are involved in the production of weapon systems.

5 As part of their strategy to monitor emerging technology in security critical and dual-use areas, the EU has supported the development of the TIM Dual-use data mining tool, which uses scientific texts, like abstracts, patents, and EU project description to map the network of dual-use relevant innovation hubs (https://knowledge4policy.ec.europa.eu/text-mining/tim-dual-use_en).

## References

[CR1] Acosta M, Coronado D, Ferrandiz E, Marin MR, Moreno PJ (2017). Patents and dual-use technology: An empirical study of the world’s largest defence companies. Defence and Peace Economics.

[CR2] Acosta M, Coronado D, Ferrándiz E, Marín MR, Moreno PJ (2019). Civil-military patents and technological knowledge flows into the leading defense firms. Armed Forces and Society.

[CR3] Acosta M, Coronado D, Marín R (2011). Potential dual-use of military technology: Does citing patents shed light on this process?. Defence and Peace Economics.

[CR4] Acosta M, Coronado D, Marín R, Prats P (2013). Factors affecting the diffusion of patented military technology in the field of weapons and ammunition. Scientometrics.

[CR5] Agrawal A, Gans J, Goldfarb A, Lerner J, Stern S (2018). Economic policy for artificial intelligence. Innovation policy and the economy.

[CR6] Arkin RC, Ulam P, Wagner AR (2012). Moral decision making in autonomous systems: Enforcement, moral emotions, dignity, trust, and deception. Proceedings of the IEEE.

[CR7] Baruffaldi S, von Beuzekom B, Dernis H, Harhoff Di, Roa N, Rosenfeld D, Squicciarini M (2020). Identifying and measuring developments in artificial intelligence: Making the impossible possible (Issue 5). OECD Publishing.

[CR82] Bouvry, P., Chaumette, S., Danoy, G., Guerrini, G., Jurquet, G., Kuwertz, A., Muller, W., Rosalie, M., & Sander, J. (2016). Using heterogeneous multilevel swarms of UAVs and high-level data fusion to support situation management in surveillance scenarios. In *2016 IEEE international conference on multisensor fusion and integration for intelligent systems (MFI)* (pp. 424–429). IEEE.

[CR8] Brundage, M., Avin, S., Clark, J., Toner, H., Eckersley, P., Garfinkel, B., Dafoe, A., Scharre, P., Zeitzoff, T., Filar, B., Anderson, H., Roff, H., Allen, G. C., Steinhardt, J., Flynn, C., Héigeartaigh, S. Ó., Beard, S., Belfield, H., Farquhar, S., … Amodei, D. (2018). The malicious use of artificial intelligence: Forecasting, prevention, and mitigation (Issue February).

[CR84] Bulatov, D., Häufel, G., Meidow, J., Pohl, M., Solbrig, P., & Wernerus, P. (2014). Context-based automatic reconstruction and texturing of 3D urban terrain for quick-response tasks. *ISPRS Journal of Photogrammetry and Remote Sensing*, *93*, 157–170.

[CR9] Cady F (2017). The data science handbook. John Wiley Sons.

[CR10] Callari, F. G., Durand, J.-G. D., Yarlagadda, P. K. K., & Glozman, T. (2021). *Techniques for managing processing resources* (United States Patent Patent No. US 10,893,107 B1). https://patentimages.storage.googleapis.com/64/43/f2/7b8b2e6efe325b/US10893107.pdf.

[CR11] Cath C (2018). Governing artificial intelligence: Ethical, legal and technical opportunities and challenges. Philosophical Transactions of the Royal Society A. Mathematical Physical and Engineering Sciences.

[CR12] Coeckelbergh M (2020). Artificial intelligence, responsibility attribution, and a relational justification of explainability. Science and Engineering Ethics.

[CR13] CPC. (2019). *G06N: Computer systems based on specific computational models*. https://www.uspto.gov/web/patents/classification/cpc/html/cpc-G06N.html.

[CR14] Cummings ML (2006). Integrating ethics in design through the value-sensitive design approach. Science and Engineering Ethics.

[CR15] Edler J, James AD (2015). Understanding the emergence of new science and technology policies: Policy entrepreneurship, agenda setting and the development of the European Framework Programme. Research Policy.

[CR16] European Commission. (2013). *Towards a more competitive and efficient European defence and security sector*. European Commission. https://ec.europa.eu/commission/presscorner/detail/en/IP_13_734.

[CR17] European Commission. (2015). *EU funding for Dual Use—A pratical guide to accessing EU funds for European Regional Authorities and SMEs*. https://ec.europa.eu/docsroom/documents/12601/attachments/1/translations.

[CR18] European Commission. (2019). *Ethics guidelines for trustworthy AI*. https://digital-strategy.ec.europa.eu/en/library/ethics-guidelines-trustworthy-ai.

[CR19] European Patent Office. (2021a). 3.3.1 Artificial intelligence and machine learning. In *Guidelines for examination*. https://www.epo.org/law-practice/legal-texts/html/guidelines/e/g_ii_3_3_1.htm.

[CR20] European Patent Office. (2021b). Part G patentability. In *Guidelines for examination*. https://www.epo.org/law-practice/legal-texts/guidelines.html.

[CR21] Evans NG (2014). Dual-use decision making: Relational and positional issues. Monash Bioethics Review.

[CR22] Favaro, M. (2021). Weapons of mass distortion: A new approach to emerging technologies, risk reductoin, and the global nuclear order.* Comunicar* (Issue May). 10.3916/c22-2004-09

[CR23] Fleurant, A., Kuimova, A., Tian, N., Wezeman, P. D., & Wezeman, S. T. (2017). The SIPRI Top 100 arms-producing and military services companies, 2016. *SIPRI Fact Sheet*, *December*, 1–8.

[CR24] Flick, U. (2014). *An introduction to qualitative research*. SAGE Publications.

[CR25] Floridi L, Cowls J, Beltrametti M, Chatila R, Chazerand P, Dignum V, Luetge C, Madelin R, Pagallo U, Rossi F, Schafer B, Valcke P, Vayena E (2018). AI4People—an ethical framework for a good AI society: Opportunities, risks, principles, and recommendations. Minds and Machines.

[CR26] Forge J (2010). A note on the definition of “dual use”. Science and Engineering Ethics.

[CR27] Fraunhofer IOSB. (2018). *Fraunhofer IOSB: Annual report 2017/2018*. https://www.energie.fraunhofer.de/content/dam/energie/en/documents/05_PDF_annual_reports/iosb_jb_2017_2018_en.pdf.

[CR28] Fraunhofer IOSB. (2020). *Fraunhofer IOSB: Business units*. https://www.iosb.fraunhofer.de/servlet/is/12576/.

[CR29] German Federal Ministry of Defense. (2017). *Military scientific research report annual report 2017: defence research for the German armed forces*.

[CR30] Gill AS (2019). Artificial intelligence and international security: The long view. Ethics and International Affairs.

[CR31] Goodfellow I, Bengio Y, Courville A (2016). Deep learning.

[CR32] Gray PS, Williamson JB, Karp DA, Dalphin JR (2007). The research imagination: An introduction to qualitative and quantitative methods. Cambridge University Press.

[CR33] Grodzinsky FS, Miller KW, Wolf MJ (2011). Developing artificial agents worthy of trust: “Would you buy a used car from this artificial agent?”. Ethics and Information Technology.

[CR34] Grunwald A (2020). The objects of technology assessment Hermeneutic extension of consequentialist reasoning. Journal of Responsible Innovation.

[CR35] Guthrie, G. (2019, December 3). Machine learning as a service (MLaaS) is the next trend no one is talking about. *DataDrivenInvestor*.

[CR36] Hagendorff T (2020). The ethics of AI ethics: An evaluation of guidelines. Minds and Machines.

[CR37] Harris, E. D. (Ed.). (2016). *Governance of dual-use technologies: Theory and practice*. American Academy of Arts & Sciences. https://www.amacad.org/sites/default/files/publication/downloads/GNF_Dual-Use-Technology.pdf.

[CR85] IJsselmuiden, J., Münch, D., Grosselfinger, A. K., Arens, M., & Stiefelhagen, R. (2014). Automatic understanding of group behavior using fuzzy temporal logic. *Journal of Ambient Intelligence and Smart Environments*, *6*(6), 623–649.

[CR38] Kim DH, Lee BK, Sohn SY (2016). Quantifying technology-industry spillover effects based on patent citation network analysis of unmanned aerial vehicle UAV. Technological Forecasting and Social Change.

[CR39] Klinger, J., Mateos-Garcia, J., & Stathoulopoulos, K. (2018). Deep learning, deep change? Mapping the development of the Artificial Intelligence General Purpose Technology. *CoRR*, *abs/1808.0*.

[CR40] Koenig N (2020). Leading beyond civilian power: Germany’s role re-conception in European crisis management. German Politics.

[CR41] Korenberg, A., & Hamer, T. (2018, December 3). *Assessing the EPO’s new guidelines on AI*. IP STARS.

[CR42] Liu W, Tao Y, Yang Z, Bi K (2019). Exploring and visualizing the patent collaboration network: A case study of smart grid field in China. Sustainability.

[CR43] Luhmann N (1979). Trust: A mechanism for the reduction of social complexity.

[CR44] Lupu M, Mayer K, Kando N, Trippe AJ, Lupu M, Mayer K, Kando N, Trippe AJ (2011). Preface. Current challenges in patent information retrieval.

[CR45] Marzi, T., Knappertsbusch, V., Marzi, A., Naumann, S., Deerberg, G., & Waidner, E. (2018). Fragen zu einer biologischen Technik. *UMSICHT-Diskurs Heft*, *2*.

[CR46] Meunier FX, Bellais R (2019). Technical systems and cross-sector knowledge diffusion: An illustration with drones. Technology Analysis and Strategic Management.

[CR47] Mowery DC, Simcoe T (2002). Is the internet a US invention? An economic and technological history of computer networking. Research Policy.

[CR48] Nissenbaum H (2001). Securing trust online: Wisdom or oxymoron?. Boston University Law Review.

[CR49] Okakita, Y. (2019). *Patent examination practices regarding AI-related inventions: Comparison in the EPO, USPTO and JPO* [MIPLC Master Thesis Series]. https://papers.ssrn.com/sol3/papers.cfm?abstract_id=3652173.

[CR50] Oltmann S (2015). Dual use research: Investigation across multiple science disciplines. Science and Engineering Ethics.

[CR51] Pecotic, A. (2019, May 3). Whoever predicts the future will win the AI arms race. *Foreign Policy*. https://foreignpolicy.com/2019/03/05/whoever-predicts-the-future-correctly-will-win-the-ai-arms-race-russia-china-united-states-artificial-intelligence-defense/.

[CR52] de Pereira, S. A. & Quoniam, L. (2017). Intellectual property and patent prospecting as a basis for knowledge and innovation: A study on mobile information technologies and virtual processes of communication and management. *RAI Revista de Administração e Inovação*. 10.1016/j.rai.2017.07.006

[CR81] Philipp, P., Schreiter, L., Giehl, J., Fischer, Y., Raczkowsky, J., Schwarz, M., Woern, H., & Beyerer, J. (2016). Situation detection for an interactive assistance in surgical interventions based on dynamic bayesian networks. *CRAS 2016, 6th joint workshop on new technologies for computer/robot assisted surgery*.

[CR53] Reppy, J. (2006). Managing dual-use technology in an age of uncertainty. *The Forum: A Journal of Applied Research in Contemporary Politics*, *4*(1).

[CR54] Riebe T, Reuter C, Reuter C (2019). Dual use and dilemmas for cybersecurity, peace and technology assessment. Information technology for peace and security: IT-applications and infrastructures in conflicts, crises, war, and peace.

[CR55] Riebe T, Schmid S, Reuter C (2020). Meaningful human control of lethal autonomous weapon system: The CCW-debate and its implications for value-sensitive design. IEEE Technology and Society Magazine.

[CR56] Riebe T, Schmid S, Reuter C (2021). Measuring spillover effects from defense to civilian sectors: A quantitative approach using linkedIn. Defence and Peace Economis.

[CR57] Roberts H, Cowls J, Morley J, Taddeo M, Wang V, Floridi L (2021). The Chinese approach to artificial intelligence: An analysis of policy, ethics, and regulation. AI and Society.

[CR58] Ronggui, H. (2019). *RQDA*. https://github.com/Ronggui/RQDA.

[CR59] Ryan M (2020). In AI we trust: Ethics, artificial intelligence, and reliability. Science and Engineering Ethics.

[CR60] Schmid J (2017). The diffusion of military technology. Defence and Peace Economics.

[CR61] Shields J (2018). Smart machines and smarter policy: Foreign investment regulation, national security, and technology transfer in the age of artificial intelligence. SSRN.

[CR62] Taddeo M (2010). Modelling trust in artificial agents, a first step toward the analysis of e-trust. Minds and Machines.

[CR63] Taddeo M (2017). Trusting digital technologies correctly. Minds and Machines.

[CR64] Taddeo M, McCutcheon T, Floridi L (2019). Trusting artificial intelligence in cybersecurity is a double-edged sword. Nature Machine Intelligence.

[CR65] Taebi B, van den Hoven J, Bird SJ (2019). The importance of ethics in modern universities of technology. Science and Engineering Ethics.

[CR66] Tavani HT (2018). Can social robots qualify for moral consideration? Reframing the question about robot rights. Information Switzerland.

[CR67] Thiebes S, Lins S, Sunyaev A (2020). Trustworthy artificial intelligence. Electronic Markets.

[CR68] Tiedrich LJ, Discher GS, Argent F, Rios D (2020). 10 Best practices for artificial intelligence-related intellectual property. Intellectual Property & Technology Law Journal.

[CR69] Tucker, J. B. (Ed.). (2012). *Innovation, dual use, security: Managing the risks of emerging biological and chemical technologies*. MIT Press.

[CR70] Umbrello S (2019). Imaginative value sensitive design: Using moral imagination theory to inform responsible technology design. Science and Engineering Ethics.

[CR71] Umbrello, S., & De Bellis, A. F. (2018). A value-sensitive design approach to intelligent agents. *Artificial Intelligence Safety and Security*, *January*, 395–410. 10.13140/RG.2.2.17162.77762

[CR72] Urquhart, Q. E., & Sullivan, L. (2020, April 27). *April 2020: The increasing importance of trade secret protection for artificial intelligence*. JD SUPRA. https://www.jdsupra.com/legalnews/april-2020-the-increasing-importance-of-64465/

[CR73] USPTO. (2019). *Cooperative patent classification: B64G cosmonautics; vehicles or equipment thereof*. https://www.uspto.gov/web/patents/classification/cpc/html/cpc-B64G.html.

[CR74] Uttley M (2019). Review of ‘the emergence of EU defense research policy: From innovation to militarization’. Defence and Peace Economics.

[CR75] Verbruggen M (2019). The role of civilian innovation in the development of lethal autonomous weapon systems. Global Policy.

[CR76] Verdiesen, I. (2017). *Agency perception and moral values related to Autonomous Weapons: An empirical study using the Value-Sensitive Design approach*. Delft University of Technology.

[CR77] Wagner AR, Arkin RC (2011). Recognizing situations that demand trust. Proceedings-IEEE international workshop on robot and human interactive communication.

[CR78] Winfield AFT, Jirotka M (2018). Ethical governance is essential to building trust in robotics and artificial intelligence systems. Philos Trans R Soc A Math Phys Eng Sci.

[CR79] WIPO. (2019). *WIPO Technology Trends 2019: Artificial intelligence*. World Intellectual Property Organization. https://www.wipo.int/edocs/pubdocs/en/wipo_pub_1055.pdf.

[CR80] Zambetti, M., Sala, R., Russo, D., Pezzotta, G., & Pinto, R. (2018). A patent review on machine learning techniques and applications: Depicting main players, relations and technology landscapes. *Proceedings of the Summer School Francesco Turco*, *2018-Septe*, 115–128.

[CR83] Ziehn, J. R., Ruf, M., Willersinn, D., Rosenhahn, B., Beyerer, J., & Gotzig, H. (2016). A tractable interaction model for trajectory planning in automated driving. In *2016 IEEE 19th international conference on intelligent transportation systems (ITSC)* (pp. 1410–1417). IEEE.

