# Supplementary figures and images for: Dual-Use and Trustworthy? A Mixed Methods Analysis of AI Diffusion Between Civilian and Defense R&D
Source: Sci Eng Ethics. 2022 Mar 8;28(2):12. doi: 10.1007/s11948-022-00364-7 (PMC8904348; doi:10.1007/s11948-022-00364-7)

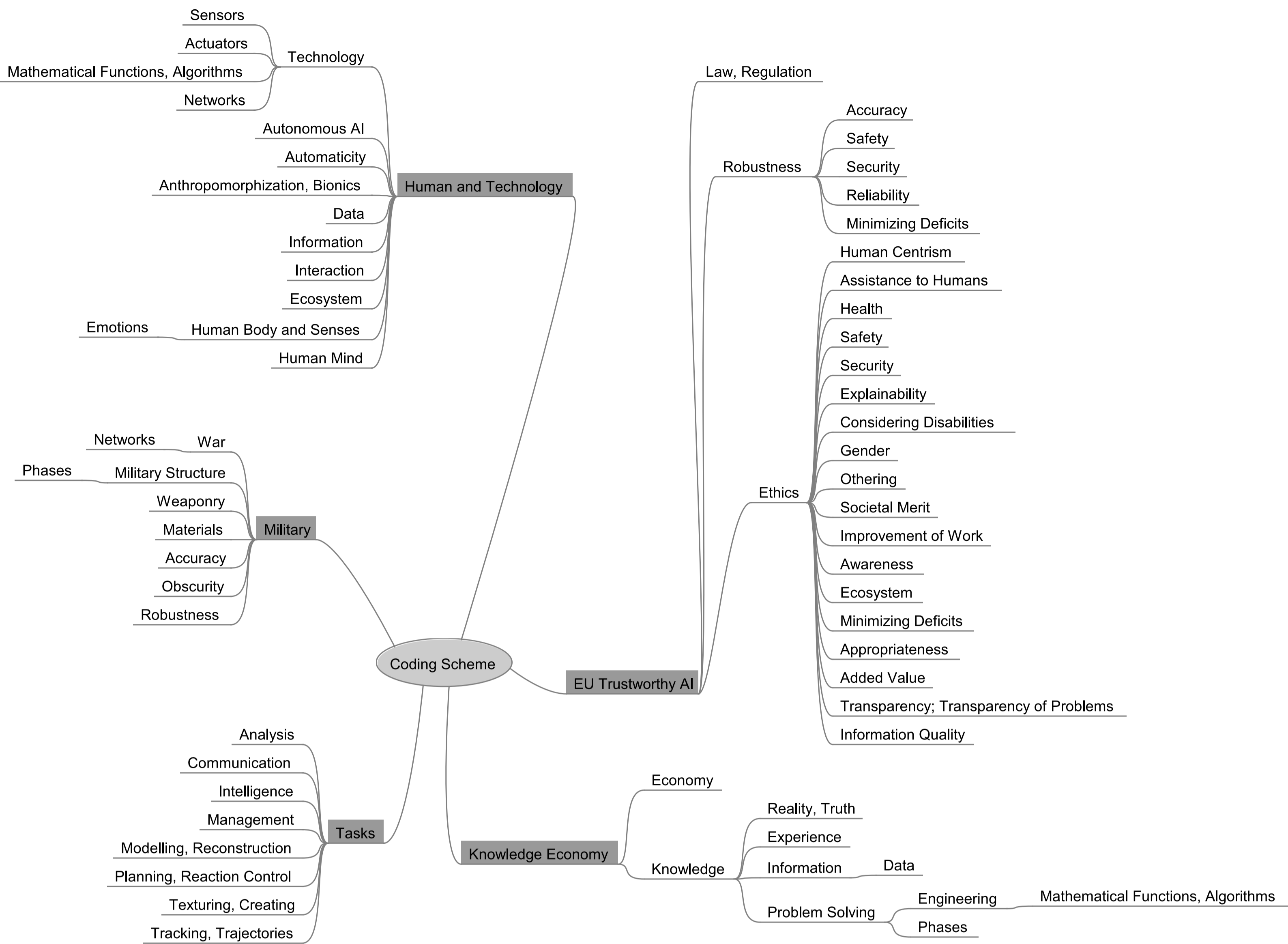

Figure A. Coding scheme for qualitative analysis

Supplement: Supplementary file 1 — (PDF 171 kb) [file 11948_2022_364_MOESM1_ESM.pdf]
