# Supplementary material for: Dual-Use and Trustworthy? A Mixed Methods Analysis of AI Diffusion Between Civilian and Defense R&D
Source: Sci Eng Ethics. 2022 Mar 8;28(2):12. doi: 10.1007/s11948-022-00364-7 (PMC8904348; doi:10.1007/s11948-022-00364-7)
Supplement: Supplementary file 3 — (PDF 174 kb) [file 11948_2022_364_MOESM3_ESM.pdf]

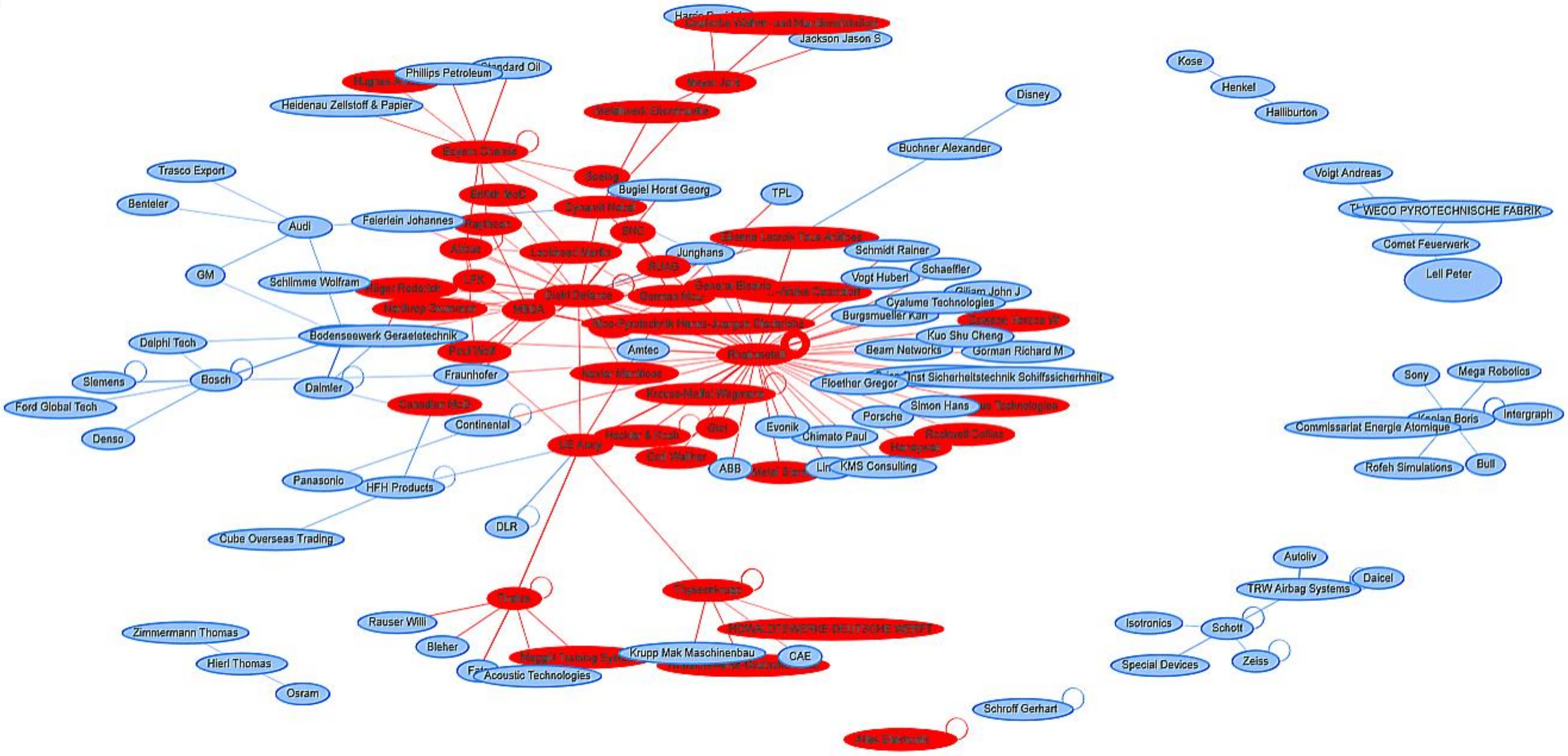

Figure C. German company network according to patent citations, with military (red) and civilian (blue) applicants. For visualization, we used [visNetwork](#) (Almende et al. 2019). Network measures were calculated with [ggraph](#) and [igraph](#) (Csárdi, 2019; Lin Pedersen, 2019).
