# Supplementary material for: Dual-Use and Trustworthy? A Mixed Methods Analysis of AI Diffusion Between Civilian and Defense R&D
Source: Sci Eng Ethics. 2022 Mar 8;28(2):12. doi: 10.1007/s11948-022-00364-7 (PMC8904348; doi:10.1007/s11948-022-00364-7)
Supplement: Supplementary file 4 — (PDF 156 kb) [file 11948_2022_364_MOESM4_ESM.pdf]

**Values**	N_{Mil} **(Word Occurrences)**	N_{Civ} **(Word Occurrences)**
Focus on data	190	96
Focus on information	77	32
Focus on input	29	23
Awareness: geography	21	0
Awareness: space	96	276
Awareness: time	65	231
Robustness	13	1
Accuracy	50	25
Obscurity	51	3
Information quality	16	11
Awareness: adaptivity	5	6
Awareness: object	38	107
Awareness: target	15	2
Awareness: movement	23	26
Explainability	7	76
Human centrism: Assistance to humans	76	601
Health	0	2
Safety, security	32	29
Intelligence of systems	26	103
Modelling, reconstruction	133	178
Automation	56	97
Autonomy	16	16
Self-consciousness, reasoning	28	97
Behavior	5	72
Gestures	1	22

Table A. Frequency of word stems representing values in civilian and military corpora, $n_{Doc}=13$
