# Supplementary material for: Dual-Use and Trustworthy? A Mixed Methods Analysis of AI Diffusion Between Civilian and Defense R&D
Source: Sci Eng Ethics. 2022 Mar 8;28(2):12. doi: 10.1007/s11948-022-00364-7 (PMC8904348; doi:10.1007/s11948-022-00364-7)
Supplement: Supplementary file 2 — (PDF 254 kb) [file 11948_2022_364_MOESM2_ESM.pdf]

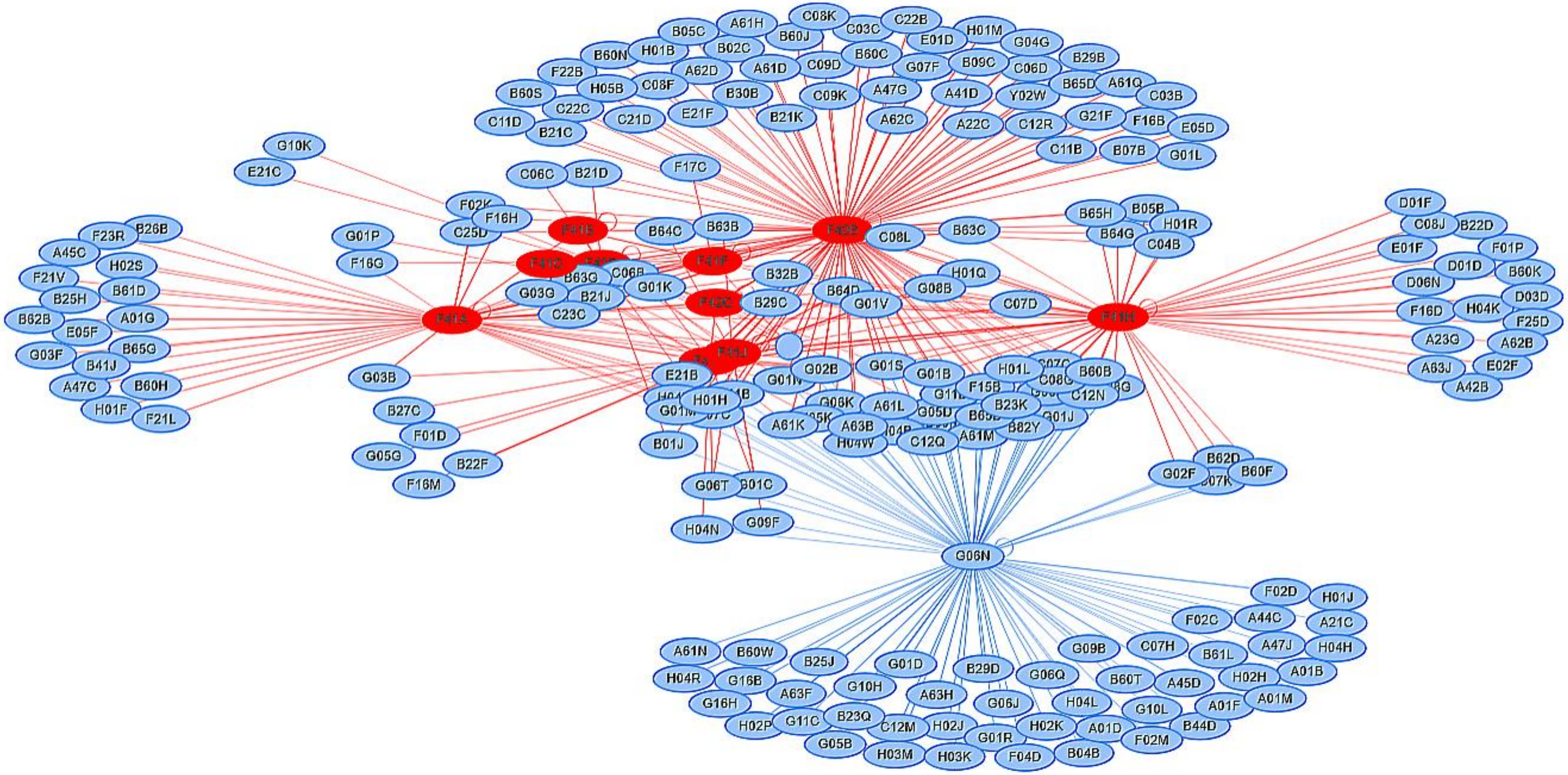

Figure B. European patent network with patent classes as nodes linked by patent citations, n=2,438. For visualization, we used visNetwork (Almende et al. 2019). Network measures were calculated with qgraph and igraph (Csárdi, 2019; Lin Pedersen, 2019).
